# Chronic Energy Deficiency and Its Determinant Factors among Adults Aged 18–59 Years in Ethiopia: A Cross-Sectional Study

**DOI:** 10.1155/2021/8850241

**Published:** 2021-01-06

**Authors:** Samuel Dagne, Yonatan Menber, Yosef Wassihun, Gedefaw Dires, Atitegeb Abera, Seteamlak Adane, Melese Linger, Zelalem T. Haile

**Affiliations:** ^1^School of Public Health, College of Medicine and Health Science, Bahir Dar University, Bahir Dar, Ethiopia; ^2^Department of Public Health, College of Health Science, Woldia University, Woldia, Ethiopia; ^3^Ohio University, Athens, USA

## Abstract

**Background:**

The prevalence of undernutrition in low- and middle-income countries is still remarkably high. Undernutrition during adulthood is a greater risk factor for low productivity, poor health, and mortality. There is limited information on the prevalence and determinants of chronic energy deficiency in Ethiopia.

**Objective:**

To assess the prevalence and determinants of chronic energy deficiency among adults aged 18–59 years in Ethiopia.

**Method:**

A secondary data analysis was conducted using the data obtained from the 2016 Ethiopia Demographic and Health Survey. Data were collected using a multistage stratified cluster sampling technique, and the analytic sample consisted of 9,280 adults aged 18–59 years. The chi-square test and multivariable logistic regression analyses were used, and *p* value <0.05 was taken as statistically significant.

**Result:**

A total of 9280 adults aged 18–59 years were included in the study and 2911 (28.7%) (95% CI: 27.0%–30.4%) of whom were chronic energy deficient. Adults who have no work (AOR = 1.41, 95% CI: 1.16, 1.72), male adults from Tigray region (AOR = 2.23, 95% CI: 1.61, 3.09), Afar region (AOR = 2.98, 95% CI: 2.04, 4.36), Somali region (AOR = 3.14, 95% CI: 2.19, 4.52), Gambella region (AOR = 1.89, 95% CI: 1.29, 2.76), Harari region (AOR = 1.54, 95% CI: 1.09, 2.19), Amhara region (AOR = 1.53, 95% CI: 1.09, 2.13), Oromia region (AOR = 1.53, 95% CI: 1.07, 2.19), Dire Dawa (AOR = 1.45, 95% CI: 1.03, 2.05), adults live lonely (AOR = 1.44, 95% CI: 1.21, 1.71), and adults residing in poor wealth index households (AOR = 1.26 : 95% CI: 1.07, 1.49) were significantly associated with chronic energy deficiency. *Conclusion and recommendation*. Chronic energy deficiency among male adults in Ethiopia was a high public health problem. Marital status, wealth index, occupation, and region were significant predictors of chronic energy deficiency. The Ministry of Health with other partners should strictly monitor and evaluate interventions that are being applied and should give focus to adult men to prevent malnutrition.

## 1. Introduction

Nowadays, low- and middle-income countries are currently attacked by a double burden of malnutrition. The rates of overweight and obesity are increasing much faster in the developing world. The prevalence of undernutrition is still remarkably high [1–4]. Chronic energy deficiency (CED) is defined as a steady-state at which a person is in energy balance, although at a “cost” either in terms of health risk or as an impairment of functions and health [5]. Chronic energy deficiency can also be defined based on body mass index (BMI) as <18.5 kg/m^2^ [6, 7].

Chronic energy deficiency during adulthood is associated with different adverse functional consequences. It increases the risk of morbidity, and mortality together with decreased mental and cognitive development reduces educational achievement and labor productivity [8–11].

The most common contributing factors to chronic energy deficiency among adults include inadequate diet intake, socioeconomic status, and infection [12–14].

Today, nearly one in three persons globally suffers from at least one form of malnutrition: wasting, stunting, vitamin and mineral deficiency, overweight or obesity, and diet-related noncommunicable diseases (NCDs). In 2003, about 7.2% of adults worldwide were underweight [3]. In 2014, about 462 million (18.5%) adults worldwide were underweight. Of this, 40.6 million (8.8%) were men. More than 20% of men in India, Bangladesh, Timor-Leste, Afghanistan, Eritrea, and Ethiopia were underweight [15].

According to the 2011 Ethiopia Demographic Health Survey (EDHS), the prevalence of male adult thinness or underweight was 28.9%. Of these, 21.4% was mild underweight and 7.3% was moderate to severe underweight [16]. The prevalence of CED was highest in the Somali region (62.0%) and lowest in Addis Ababa (22.0%) [16]. In Ethiopia, the prevalence of male adult CED increased from 10.8% in 2003 to 25.3% in 2011 [3, 16].

The United Nations set the sustainable development goal (SDG) targets a reduction in a different form of malnutrition by 2030 [17–19]. The World Health Organization (WHO) also sets a policy brief called double-duty actions for nutrition to end all burdens of malnutrition by 2030 [20].

The Ethiopian government identified different forms of undernutrition in the selected group such as children, adolescents, pregnant, and lactating women as the key priority indicators for national development. Besides global initiatives, the Ethiopian government had exerted many efforts, and bold actions were taken in health and other nutrition-sensitive and specific interventions. The Seqota declaration reflects the commitment to ending undernutrition by 2030 using the multisectoral National Nutrition Program (NNP-II) as a guiding framework to achieve this goal [19, 21–24].

Although there is limited documented data on male adult CED in Ethiopia, available sources documented increments in the prevalence of CED from 10.8% to 28.9% between 2003 and 2011 [3, 16]. However, almost all nutrition-related policies, strategies, and programs in Ethiopia give priority to undernutrition among special target groups such as children, adolescents, pregnant, and lactating. To some extent, little attention is also now given to the current emerging adult obesity. But, chronic energy deficiency among male adults is a forgotten agenda in Ethiopia. Moreover, lifecycle malnutrition can be tackled by intervening at all stages of life and all forms of malnutrition.

At the same time in Ethiopia, though there is too huge available data on published literature on undernutrition and concurrent morbidity, studies on CED among male adults are too limited. Finding from this study also helps to draw conclusions and to translate findings into policies and practices. Therefore, this study aimed to investigate the prevalence and determinant factors of CED among adults aged 18–59 years old in Ethiopia.

### 1.1. Study Design and Setting

Secondary data analysis was conducted using the data obtained from 2016 EDHS. EDHS was a cross-sectional community-based study that was conducted from January to June 2016. A detailed description of study design and methods for the 2016 EDHS is available elsewhere [25]. In brief, a two-stage stratified cluster sampling technique was used using the 2007 Ethiopia Population and Housing Census as the sampling frame. In the first stage, 645 enumeration areas (EA) were selected using probability proportional to the EA size. In the second stage, 28 households per EA were selected with an equal probability of systematic selection. Eligible participants included all adult men aged between 18 and 59 years. Additionally, in all selected households, weight and height measurements were collected from male adults aged 18–59 years (*n* = 11, 100). After excluding adult male participants who did not have a response to the outcome variable, we restricted our analytical sample to 9,280 males aged 18–59 years.

## 2. Measurement

### 2.1. Dependent Variable

The nutritional status of adult men was screened by measuring height and weight and calculating BMI. The height of adult men aged 18–59 years was measured using a height scale. The men standing upright with their barefoot and the men's heads, shoulders, buttocks, knees, and heels were made to touch the height scale. The reading was recorded to the nearest 0.1 cm. The weight of study participants was measured with minimum/light/clothing and no shoes with the reading recorded to the nearest 0.1 kg [26]. BMI was calculated by dividing weight in kilograms by height in meters squared (kg/m^2^). Adult men with a BMI of less than 18.5 kg/m^2^ were considered as exhibiting chronic energy deficiency [5, 27]. The body mass index (BMI) values of less than 16, 16–16.99, and 17–18.4 were used to classified adults as severe, moderate, and mild CED, respectively. BMI of 18.5–24.9 was classified as normal weight [6].

### 2.2. Independent Variables

Based on existing literature, the following covariates were selected: anemia, age, household head sex, number of children in the household, family size, education level, occupational status, marital status, and place of residence, religion, household wealth index, region, alcohol intake, and chat chewing. Anemia in adults was defined based on the WHO recommendation, and hemoglobin level <13 g/dl was categorized as anemic [6]. The household wealth index was computed using principal components analysis on the household asset and was categorized into five wealth quintiles (lowest, second, middle, fourth, and highest) [28]. In this study, lowest and second wealth quintiles were grouped to poor, while the fourth and highest wealth quintiles were grouped to rich. A detailed description of the calculation of the household wealth index is available elsewhere [25].

### 2.3. Data Management and Statistical Analysis

Data were extracted from EDHS 2016, and further coding and analysis were performed using SPSS version 20. Throughout the analysis, sample weights were carried out to adjust for nonproportional allocation of the sample to strata and regions during the survey process and to restore the representativeness. Descriptive statistics were conducted to provide a summary of the characteristics of the study sample. Bivariate analysis with chi-square statistics was performed to test the independence of distribution between the independent variables and chronic energy deficiency. A multiple logistic regression model was then fitted to identify the determinants of chronic energy deficiency. To control the confounding effect, all variables with a *p* value less than 0.25 in the bivariate analysis were included in the final regression model. In the final model, variables with a *p* value less than 0.05 were considered as significantly associated with CED. The corresponding odds ratio with 95% confidence intervals was reported. Sampling weights that accounted for complex survey design were incorporated in all analyses. All statistical analyses were conducted using SPSS version 20.0.

## 3. Results

Table 1 presents the characteristics of the study population. Most of the households, 5664 (64.9%), had five and more family sizes. About 3709 (44.0%) adults had a primary education level. The majority of study participants, 6874 (83.8%), were rural residents. About 3661 (35.95%) adults reside in poor wealth index households. About 4304 (48.3%) study subjects had a history of alcohol intake (Table 1).

**Figure 1:**
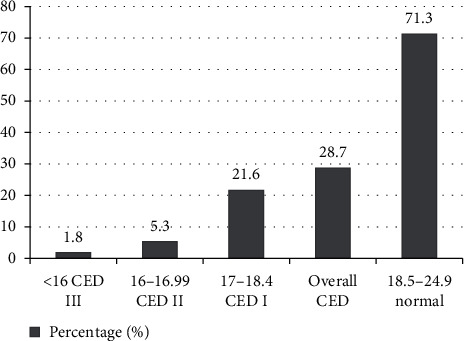
Nutritional status of adults aged 18–59 years in Ethiopia: EDHS 2016 (*N* = 9280).

The nutritional status of subjects is shown in Figure 1. The mean height, weight, and body mass index (BMI) of the subjects were 168·5 cm, 55.7 kg, and 19·6 kg/m^2^, respectively. The overall frequencies of CED (BMI < 18·5 kg/m^2^) was 28.7% (95% CI: 27.0%–30.4%). About 1.8% and 5.8% of subjects had severe and moderate CED, respectively. Based on the WHO (1995) classification, the prevalence of CED among this population was high (20–39%), and thus, the situation is a serious public health problem.

Differences in the proportions of chronic energy deficiency by participant characteristics are presented in Table 2. The prevalence of CED was higher among households with ≥5 family size than households with <5 family members (*p* < 0.001). The proportion of CED was higher among households with a poor wealth index than households with a high wealth index. Factors that were associated with CED included anemia, sex of head of household, educational status, place of residence, religion, wealth status, marital status, occupational status, adults age, family size, alcohol consumption, and region (Table 2).

**Figure 2:**
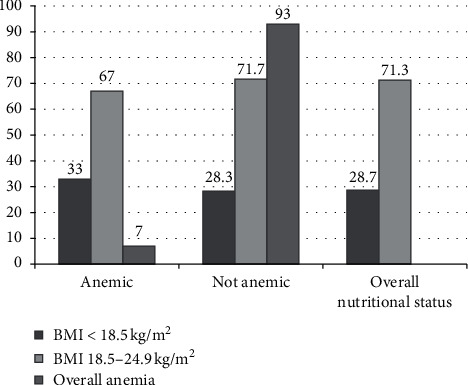
Chronic energy deficiency anemia comorbidity among adults aged 18–59 years old in Ethiopia: EDHS 2016 (*n* = 9280).

Chronic energy deficiency and anemia comorbidity are shown in Figure 2. A higher proportion of chronic energy deficiency was observed in anemic (33.0%) male adults than nonanemic (28.3%) adults.

Table 3 presents the multivariable-adjusted association between factors and CED.

In the bivariate regression analysis, anemia, educational status, place of residence, religion, wealth status, marital status, occupational status, adults age, alcohol consumption, and region had a *p* value <0.05. In the multivariable analysis, only occupation, marital status, household wealth index, and region were independent predictors of CED at a *p* value <0.05.

Compared to adults who have work, the odds of CED were higher in adults who have no work (95% CI: 1.23, 1.82). Compared to adults who have married, the odds of CED were higher in adults who live lonely (95% CI: 1.21, 1.71). Male adults who lived in Tigray region were 2.2 times (AOR = 2.23, 95% CI: 1.61, 3.09), Afar region were 2.9 times (AOR = 2.98, 95% CI: 2.04, 4.36), Amhara region were 1.5 times (AOR = 1.53, 95% CI; 1.09, 2.13), Oromia region were 1.5 times (AOR = 1.53, 95% CI: 1.07, 2.19), Somali region were 3.1 times (AOR = 3.14, 95% CI: 2.19, 4.52), Gambella region were 1.9 times (AOR = 1.89, 95% CI: 1.29, 2.76), Harari region were 1.5 times (AOR = 1.54, 95% CI: 1.09, 2.19), and Diredawa town were 1.5 times (AOR = 1.54, 95% CI: 1.03, 2.05) more likely to be chronic energy deficient compared to adults from Addis Ababa, respectively.

The likelihood of chronic energy deficiency was higher among adults residing in poor wealth index households than adults residing in rich wealth index households (AOR = 1.25; 95% CI: 1.05, 1.48) (Table 3).

## 4. Discussion

Chronic energy deficiency among adults is one of the abandoned nutritional problems and is one of the contributors to morbidity and mortality together with decreased mental and cognitive development. It also reduces educational achievement and labor productivity [8–10, 29–31]. The prevalence of CED among adults in Ethiopia remains significantly high. The development of effective interventions aimed at reducing rates of undernutrition requires the identification of key risk factors of CED [2, 4, 20].

In a representative sample of Ethiopian adults aged 18–59 years, we found that the overall prevalence of CED was 28.7%. Marital status, occupation, household wealth index, and region were significantly associated with chronic energy deficiency.

The prevalence of CED in this study was higher than from study performed in Uganda (22.3%) [32], India (19.5%) [33], Botswana (19.5%) [14], and Tharu population, India (26.2%) [34], Malaysia 8.5% [35], and Colombia (2.8%) [36]. However, it was lower than the study report from west India (40.1%) [37]. These discrepancies might be due to sampling size, socioeconomic, and feeding habit differences between study setups.

The odds of CED among adult men who lived lonely were higher compared with married adult men. This might be explained by married adult men unlike adult lived lonely counterparts are more likely to be younger; they have a high risk of being underweight due to low intake of diet and dependency on the family. This finding is similar to many studies conducted elsewhere [14, 32]. Marriages mostly expose adults to be overweight/obese [38–40].

Compared to men who have work, the odds of CED were higher in adults who have no work. This might be explained by those men who have no work may not get enough money for food. The other possible justification could be because men with no work mostly consume less nutritious food which exposed them to CED. This finding is similar to many studies conducted elsewhere [41–44].

The likelihood of chronic energy deficiency was higher among adults residing in poor wealth index households than adults residing in rich wealth index households. Similar findings were reported from studies conducted elsewhere [3, 14, 29, 41, 45]. This could be due to those poorer households that are unable to purchase nutritionally adequate and diversified food for their family. This leads to inadequate food intake, exposure to infections, and lack of access to basic health services.

The odds of CED among Somali, Afar, Tigri, Gambella, Benishangul-Gumuz, Harari, Oromia, Amahara, and Dire Dawa adult men were 3.1, 2.9, 2.2, 1.9,1.6, 1.5, 1.5, 1.5, and 1.5 times higher than adults from Addis Ababa, respectively. The possible hypothesis could be feeding habits and the type of food production difference in the northeastern parts of Ethiopia.

The main strength of this study was that it used nationally representative data with a large sample size which could enhance the generalisability of the findings. However, this study had limitations that the EDHS survey is relied on respondents' self-report and might have the possibility of recall bias and social desirability bias as data were collected by a self-reported interview. Again, this study is a cross-sectional study design, and it is difficult to establish causality between the outcome of interest and these important independent variables.

## 5. Conclusion and Recommendation

Chronic energy deficiency among male adults in Ethiopia was a high public health problem. Marital status, wealth index, occupation, and region were significant predictors of chronic energy deficiency. The Ministry of Health with other partners should strictly monitor and evaluate interventions that are being applied and should give focus to adult men to prevent malnutrition.

## Figures and Tables

**Table 1:** Descriptive statistics of the study sample (*N* = 9280).

Characteristics	Overall, *n* (wt.%)
Family size	
<5	3616 (35.1)
≥5	5664 (64.9)

Number of children in the household	
<5	7051 (74.5)
≥5	2229 (25.5)

Anemia	
Yes	694 (7.0)
No	8586 (93.0)

Sex of household head	
Male	8035 (89.2)
Female	1245 (10.8)

Age	
18–29	4297 (44.4)
30–44	3390 (37.5)
45–59	1593 (18.0)

Educational status	
No education	2949 (34.2)
Primary	3709 (44.0)
Secondary	1519 (13.9)
Higher	1103 (8.0)

Occupational status	
Not working	1233 (8.4)
Working	8047 (91.6)

Marital status of the respondent	
Married	5845 (63.7)
Others^*∗*^	3435 (36.3)

Place of residence	
Urban	2406 (16.2)
Rural	6874 (83.8)

Religion of respondent	
Orthodox	4020 (45.2)
Catholic	68 (0.7)
Protestant	1638 (22.0)
Muslim	3462 (31.0)
Traditional	27 (0.3)
Others	65 (0.9)

Household wealth index	
Poor	3661 (35.9)
Middle	1383 (20.2)
Rich	4236 (43.9)

Region	
Tigray	979 (6.3)
Afar	515 (0.6)
Amhara	1403 (27.5)
Oromia	1304 (36.5)
Somalia	710 (2.3)
Benishangul	728 (0.9)
SNNPR	1271 (21.0)
Gambella	655 (0.3)
Harari	420 (0.2)
Dire Dawa	558 (0.5)
Addis Ababa	737 (3.8)

Alcohol intake	
Yes	4304 (48.3)
No	4976 (51.7)

Chat chewing	
Yes	3002 (28.6)
No	6278 (71.4)

Chronic energy deficiency	
Yes	2911 (28.7)
No	6369 (71.3)

^*∗*^Separated, widowed, divorced, living with a partner, and single. SNNPR, Southern Nations, Nationalities, and Peoples' Region. Weighted percentages were obtained to control for complex sample design.

**Table 2:** Characteristics of the study sample by chronic energy deficiency (*N* = 9280).

Characteristics	Chronic energy deficiency		*p*
	Yes, *n* (wt.%)	No, *n* (wt.%)	
Family size			
<5	1040 (27.4)	2576 (72.6)	<0.001
≥5	1871 (29.3)	3793 (70.7)	

Number of children in the household			
<5	2227 (29.4)	4824 (70.6)	0.425
≥5	684 (26.5)	1545 (73.5)	

Anemia			
Yes	272 (33.0)	422 (67.0)	<0.001
No	2639 (28.3)	5947 (71.7)	

Sex of household head			
Male	2477 (28.4)	5558 (71.6)	0.004
Female	434 (30.9)	811 (69.1)	

Age			
18–29	1514 (32.7)	2783 (67.3)	<0.001
30–44	925 (24.5)	2465 (75.5)	
45–59	472 (27.3)	1121 (72.7)	

Educational status			
No education	1019 (29.4)	1930 (70.6)	<0.001
Primary	1153 (29.3)	2556 (70.7)	
Secondary	469 (28.5)	1050 (71.5)	
Higher	270 (22.3)	833 (77.7)	

Occupational status			
Not working	549 (40.3)	684 (59.7)	<0.001
Working	2362 (27.6)	5685 (72.4)	

Marital status of the respondent			
Married	1679 (25.6)	4166 (74.4)	<0.001
Others	1232 (34.1)	2203 (65.9)	

Place of residence			
Urban	578 (22.8)	1828 (77.2)	<0.001
Rural	2333 (29.8)	4541 (70.2)	

Religion of respondent			
Orthodox	1095 (27.2)	2925 (72.8)	<0.001
Catholic	33 (51.4)	35 (48.6)	
Protestant	479 (27.2)	1159 (72.8)	
Muslim	1278 (31.6)	2184 (68.4)	
Traditional	7 (23.6)	20 (76.4)	
Others	19 (21.2)	46 (78.8)	

Household wealth index			
Poor	1430 (33.0)	2231 (67.0)	<0.001
Middle	413 (27.6)	970 (72.4)	
Rich	1068 (25.6)	3168 (74.4)	

Region			
Tigray	376 (37.8)	603 (62.2)	<0.001
Afar	263 (46.7)	252 (53.3)	
Amhara	388 (27.8)	1015 (72.2)	
Oromia	394 (29.4)	910 (70.6)	
Somalia	345 (48.8)	365 (51.6)	
Benishangul	201 (29.0)	527 (71.0)	
SNNPR	311 (25.0)	960 (75.0)	
Gambella	236 (31.6)	419 (68.4)	
Harari	114 (27.5)	306 (72.5)	
Dire Dawa	154 (27.1)	404 (72.9)	
Addis Ababa	129 (17.4)	608 (82.6)	

Alcohol intake			
Yes	1162 (26.5)	3142 (73.5)	<0.001
No	1749 (30.7)	3227 (69.3)	

Chat chewing			
Yes	961 (30.0)	2041 (70.0)	0.355
No	1950 (28.1)	4328 (71.9)	

**Table 3:** Factors associated with chronic energy deficiency among adults aged 18–59 years in Ethiopia: EDHS 2016 (*N* = 9280).

	Unadjusted OR (95% CI)	*p*	^*∗*^Adjusted OR (95% CI)	*p*
Family size				
<5	0.91 (0.79, 1.04)	0.181	0.95 (0.82, 1.10)	0.524
≥5	Reference		Reference	

Anemia				
Yes	1.23 (1.05, 1.55)	0.045	1.17 (0.93, 1.48)	0.163
No	Reference		Reference	

Sex of household head				
Male	0.88 (0.72, 1.09)	0.254	1.09 (0.87, 1.36)	0.437
Female	Reference		Reference	

Age				
18–29	1.29 (1.08, 1.54)	0.006	1.09 (0.87, 1.37)	0.438
30–44	0.86 (0.71, 1.05)	0.142	0.87 (0.71, 1.06)	0.164
45–59	Reference		Reference	

Education status				
No education	1.44 (1.12, 1.86)	0.004	1.25 (0.91, 1.71)	0.169
Primary	1.44 (1.12, 1.87)	0.006	1.21 (0.90, 1.61)	0.207
Secondary	1.39 (1.04, 1.84)	0.024	1.11 (0.82, 1.48)	0.515
Higher	Reference		Reference	

Occupation status				
Not working	1.77 (1.48, 2.11)	<0.001	1.49 (1.23, 1.82)	0.001
Working	Reference		Reference	

Marital status				
Married	Reference	<0.001	Reference	<0.001
Others^a^	1.51 (1.33, 1.71)		1.44 (1.21, 1.71)	

Place of residence				
Urban	0.69 (0.56, 0.86)	0.001	0.79 (0.58, 1.06)	0.122
Rural	Reference		Reference	

Household wealth index				
Poor	1.43 (1.24, 1.65)	<0.001	1.25 (1.06, 1.48)	0.009
Middle	1.11 (0.91, 1.36)	0.309	1.03 (0.83, 1.28)	0.757
Rich	Reference		Reference	

Region				
Tigray	2.89 (2.27, 3.67)	<0.001	2.23 (1.61, 3.09)	<0.001
Afar	4.17 (3.02, 5.75)	<0.001	2.98 (2.04, 4.36)	<0.001
Amhara	1.83 (1.43, 2.35)	<0.001	1.53 (1.09, 2.13)	0.012
Oromia	1.98 (1.53, 2.57)	<0.001	1.53 (1.07, 2.19)	0.021
Somalia	4.45 (3.38, 5.87)	<0.001	3.14 (2.19, 4.52)	<0.001
Benishangul-Gumuz	1.95 (1.42, 2.67)	<0.001	1.58 (1.06, 2.35)	0.021
SNNPR	1.59 (1.22, 2.07)	0.001	1.26 (0.87, 1.82)	0.211
Gambella	2.19 (1.56, 3.08)	<0.001	1.89 (1.29, 2.76)	0.001
Harari	1.80 (1.33, 2.45)	<0.001	1.54 (1.09, 2.19)	0.014
Dire Dawa	1.77 (1.29, 2.43)	<0.001	1.45 (1.03, 2.05)	0.033
Addis Ababa	Reference		Reference	

Alcohol intake				
Yes	Reference	0.006	Reference	0.318
No	1.23 (1.06, 1.43)		0.91 (0.75, 1.10)	

OR, odds ratio; CI, confidence interval, SNNPR, Southern Nations, Nationalities, and Peoples' Region; MDDS, minimum dietary diversity score. ^a^Widowed, separated, divorced, and living with a partner. ^*∗*^The final regression model was adjusted for anemia, educational status, place of residence, wealth status, marital status, occupational status, adults' age, family size, alcohol consumption, and region.

## Data Availability

The DHS data analyzed during the current study are available in the repository (https://dhsprogram.com/data).

## Abbreviations

AOR: Adjusted odd ratio
BMI: Body mass index
CED: Chronic energy deficiency
CSA: Central statistics agency
CI: Confidence interval
EDHS: Ethiopian Demographic Health Survey
NCDs: Noncommunicable diseases
NNP: National Nutrition Program
SDG: Sustainable development goal
WHO: World Health Organization.
